# Hybrid Dissection for Neutron Tube Shell via Continuous-Wave Laser and Ultra-Short Pulse Laser

**DOI:** 10.3390/mi13030352

**Published:** 2022-02-23

**Authors:** Minqiang Kang, Yongfa Qiang, Canlin Zhu, Xiangjun Xiang, Dandan Zhou, Zhitao Peng, Xudong Xie, Qihua Zhu

**Affiliations:** 1 Laser Fusion Research Center, China Academy of Engineering Physics, Mianyang 621900, China; yfqiang@zju.edu.cn (Y.Q.); 13141317443@163.com (C.Z.); dennis55555@163.com (X.X.); dan723@126.com (D.Z.); peng_zhitao@163.com (Z.P.); 2 Graduate School of China Academy of Engineering Physics, Beijing 100088, China

**Keywords:** laser cutting, non-contact process, neutron tube shells, 304 stainless steel, fiber laser, femtosecond laser, thermal transmission simulation

## Abstract

The sealed neutron tube shell dissection process utilizing the traditional lathe turning method suffers from low efficiency and high cost due to the frequency of replacement of the diamond knife. In this study, a hybrid dissection method is introduced by combining the continuous-wave (CW) laser for efficient tangential groove production with an ultra-short pulse laser for delamination scanning removal. In this method, a high-power CW laser is firstly employed to make a tapered groove on the shell’s surface, and then a femtosecond pulse laser is used to micromachine the groove in order to obtain a cutting kerf. The thermal field was theoretically investigated in a finite element model. The simulation results show that the width of the area of temperature exceeding 100 °C is 1.9 mm and 0.4 mm with rotating speeds of 20 rad/s and 60 rad/s, respectively. In addition, a 2 mm deep slot in the 25 mm diameter tube was successfully produced in 1 min by a kilowatt fiber laser, and a 500-femtosecond pulse laser was employed to cut a plate with a material removal rate of 0.2 mm^3^/min. By using the hybrid method, the cutting efficiency was improved about 49 times compared to the femtosecond laser cutting. According to the simulation and experimental results, this method provides a high-efficiency and non-thermal cutting technique for reclaimed metallic neutron tube shells with millimeter-level thick walls, which has the advantages of non-contact, minimal thermal diffusion, and no effect of molten slag. It is indicated that the hybrid dissection method not only offers a new solution for thick neutron tube shell cutting but also extends the application of laser cutting techniques.

## 1. Introduction

The neutron tubes based on ^2^H(d,n)^3^He(D-D) or ^3^H(d,n)^4^He(D-T) fusion reactions to generate monochromatic neutrons have been widely applied in the fields of neutron radiography [1,2,3], borehole logging [4], coal analysis [5], searching for water [6], etc. Typically, the neutron tube components are the ion source, acceleration electrode, hydrogen-absorbing filament, ion target, and tube shell [7,8]. The deuterium (D) and tritium (T) ions extracted from the ion source are firstly accelerated by high voltage electric field, and then focused and bombarded to the ion target, finally producing high energy neutrons. The ion target is one of the most important components, where the D and T atoms are stored, and the D-D and D-T nuclear reactions occur, and it requires a normal toleration temperature of less than 150 °C [9,10]. The neutron tube is an accelerator vacuum system with all the inside objects sealed in a tube shell made of ceramic or stainless steel [11]. Due to the massive application and limited lifetime, there is a large number of trash sealed neutron tubes which need to be reclaimed to gather back the D and T atoms. The dissection of the tube shell is the key procedure to take out the ion target. However, in order to avoid the actions of D and T ions, the safe temperature for the dissection process is required to be no more than 100 °C. During the dissection, the inner temperature is required to be lower than 50 °C and the yield molten slag is not allowed to synchronously enter the shell. In addition, it would be ideal to carry out the total dissection process in a vacuum vessel and without connection with the outside devices.

At present, the mechanical cutting method using a lathe turning process is employed to achieve the dissection of neutron tube shells. There is no coolant liquid in the diamond turning process to avoid troublesome liquid waste disposal. However, a lack of cooling makes diamond knives vulnerable to damage. Frequent replacement of diamond knives results in a low operating efficiency and high cost. Therefore, it is necessary to find a new cutting method for the effective cold cutting of a large number of waste neutron tubes. The abrasive-waterjet cutting method is a highly effective process with the absence of thermal diffusion, high machining versatility, and small machining force [12,13]. It is known that the process uses a jet of high pressure and velocity water and an abrasive slurry to cut the target material by means of erosion, and it needs a great deal of water and abrasives to finish the cutting process. Furthermore, the process is messy and characterized by huge quality variation in terms of the kerf width and striations. Obviously, it is not an appropriate approach for the dissection of neutron tube shells.

Compared with tradition methods, the laser ablation cutting process is an alternative approach to cut stainless steel plates and tubes, with the advantages of high efficiency, non-contact, low maintenance, and remote controllable [14,15]. It is known that the laser cutting of thick work-pieces provides considerable advantages over the conventional techniques owing to the short processing time and high precision operation. In addition, the flexibility of fiber lasers provides a fast and affordable way to accomplish larger work stations and more complex applications [16,17]. Nowadays, pipe sheet cutting systems equipped with a high-power fiber laser are available due to their high efficiency and reduced maintenance costs. However, in the conventional laser cutting method, the laser cutting head is usually pointed perpendicularly to the material surface and the cutting gas flows coaxially to the beam [18,19]. Moreover, the molten mass ejected from the kerf by the pressure of the cutting gas flow and the residual laser beam directed into the tube shell will both cause the temperature to rise inside the tube shell. Unlike the laser melt ablation process, ultra-short pulse laser micromachining provides a non-thermal method, utilizing high energy pulses of picosecond to femtosecond durations (e.g., <10 ps) [20,21,22]. With the effect of megawatt pulse peak power, the material is locally vaporized with minimal thermal diffusion and without any molten slag produced. However, ultra-short laser cutting is mainly used for machining ultra-thin materials with a thickness less than 300 μm [23,24]. When working with millimeter-level thickness sheets, it is not an appropriate approach due to the low removal efficiency and beam clipping by cut wall shadowing. Additionally, as the result of the obvious threshold effect and the shielding effect of plasma, the improvement of process efficiency is limited by increasing the single pulse energy and repetition frequency.

In a word, the high-power laser cutting process is highly efficient but with a large thermal diffusion region, and the ultra-short pulse micromachining process is a non-thermal technique but with ultra-low material removal rates: both of them are not suitable for the dissection of neutron tube shells. Therefore, a potential approach is to utilize the useable merits and to avoid the weaknesses of the two methods to finish the whole process. In this paper, by combining continuous-wave laser efficient tangential grooving with ultra-short pulse laser delamination scanning removal, a new dual laser beam asynchronous cutting method is presented for the efficient dissection of neutron tube shells with thick metallic walls. A three-dimensional finite element model (FEM) was established to validate the temperature control of the shell surface during the continuous-wave (CW) laser dissection process. Additionally, a kilowatt continuous-wave fiber laser and a femtosecond pulse laser were employed to process a tube part and plate sheet in the experiments to certify the novel method.

## 2. Materials and Methods

**Figure 1:**
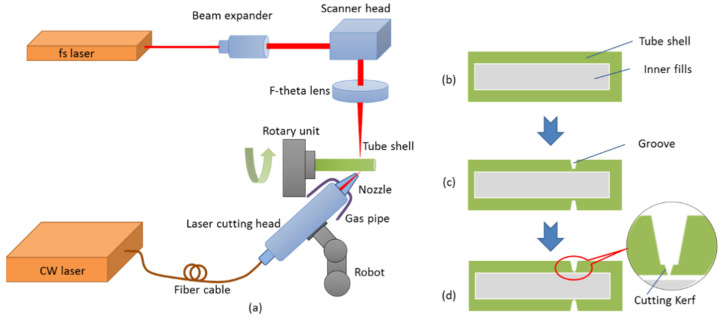
Configuration of the dual laser beam asynchronous dissection process: (**a**) schematic diagram of the cutting system, (**b**) cross-sectional view of the tube shell, (**c**) the tapered groove created by the fiber laser, and (**d**) the cutting kerf after the femtosecond laser completed cutting.

Figure 1 shows the configuration of the hybrid dissection process in this study. As shown in Figure 1a, the cutting system includes a high-power fiber laser (MFSC-1000 L, produced by MAX photonics Co., Ltd., Shenzhen, China) with a maximum output power of 1 kW at the wavelength of 1080 nm, and a homemade 7 W femtosecond pulse laser with the condition of 500 fs at 1030 nm. The CW laser beam is coupled to a laser cutting head and focused to the side direction of the tube to create a tapered groove, while the femtosecond pulse laser is passed through a beam expander, and then sent to a scanner head, and focused to the bottom of the tapered groove from the vertical direction by an F-theta lens to complete the dissection process. The tube shell is held by a rotating chuck to avoid deflections during the process, and then is fixed to a rotary unit, which maintains a controllable high turning speed movement.

### 2.1. Cutting Method

**Figure 2:**
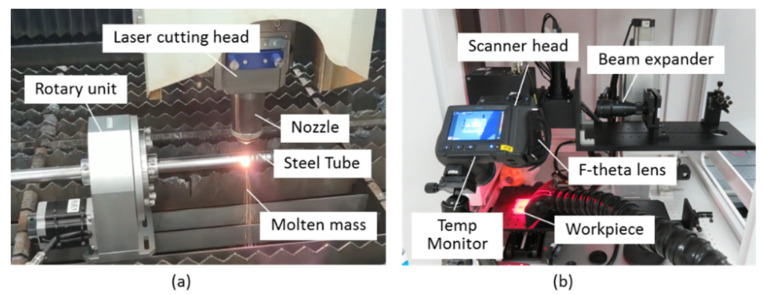
Experimental setup: (**a**) the first step: kilowatt fiber laser cutting; (**b**) the second step: femtosecond pulse laser cutting.

The experiment setup is shown in Figure 2. The hybrid dissection process includes two steps. Firstly, the tapered groove operation is created by a high-power CW fiber laser using laser ablation, which is analogous to the lathe turning process, and the laser beam assumes the action of the turning knife, as shown in Figure 2a. The groove is machined by the movement of the laser beam and the synchronous rotation of the tube shell. Then, a delamination scanning removal methodology is applied to the cutting kerf operation, which is shown in Figure 2b. The cutting kerf is machined by the scanning femtosecond pulse laser and the tube shell’s rotary, cooperatively. The temperature distribution is measured by a temperature monitoring device (infrared camera FLIR T620, produced by FLIR Systems Inc., Wilsonville, OR, USA). The whole cutting process holds a low temperature and does not lead to molten slag and residual laser directed at the inner substance. Table 1 indicates the main parameters of the two lasers used in this research.

**Figure 3:**
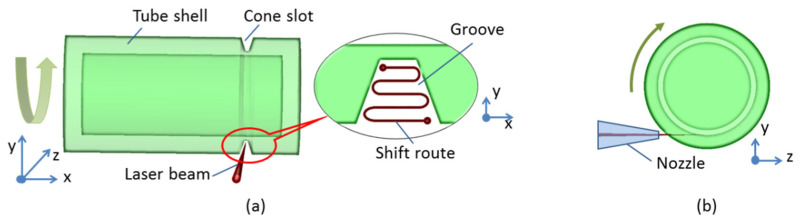
Configuration of the high-power fiber laser process: (**a**) schematic representation of laser beam action and drawing of the cut geometry; (**b**) cross-section view.

The configuration of the high-power fiber laser fabrication is shown in Figure 3. The laser beam, with an average power of 1 kW, is fed via a process fiber (50 μm core diameter) into the laser cutting head fitted with a 75 mm collimator and a 150 mm focal lens, which results in a final theoretical spot size of 100 μm. The focused laser beam passes through the nozzle tip and irradiates the tangential direction surface of the tube shell, as shown in Figure 3b. The standoff distance between the conical nozzle and the working point is set to a fixed value to avoid the space interference. The laser cutting head contained a cylindrical tank connected to two flexible gas pipe lines. The assistive argon gas with a gauge pressure of approximately 10 bar is released into the cylindrical tank and passes through the nozzle tip toward the tube shell surface. The high-pressure gas flow is used to blow away the molten metal and accelerate fume dispersion, while cooling the shell body. The laser cutting head is mounted on a robot arm device, which is controlled via the computerized numerical control, to obtain X-Y-Z stage movement of the fiber laser beam. The tube shell is held by a rotating chuck and fixed to a rotary unit, which maintains a controllable turning movement. Usually, the kerf width of fiber laser melt cutting is only equal to the focus spot diameter [15]. With the effect of the laser beam’s movement in the X-Y dimension (shown in Figure 3a), the blow down of argon gas flow, and the tube’s rotary operation, the groove is created efficiently. Additionally, as a result of the cooling effect and the short time of operation, the whole tube shell is within a small thermal impact zone and holds a low temperature status during the fast-cutting procedures. This method avoids direct illumination by the high-power laser beam and the ejection of molten mass into the inner region. Additionally, the residual thin metallic wall prevents the molten metal and features from getting into the tube shell.

**Figure 4:**
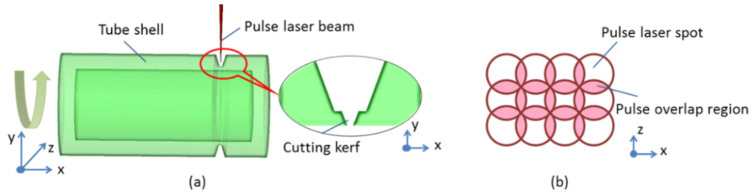
Configuration of the femtosecond pulse laser process: (**a**) schematic diagram of laser beam action; (**b**) schematic description of the scanning pattern drawn.

The schematic diagram of the femtosecond pulse laser fabrication is represented in Figure 4. The femtosecond pulse laser is passed through a beam expander to magnify the diameter of the laser spot before entering into the scanner head. Additionally, the diameter of the entrance spot is expanded beyond that of the smaller focused spot. With the use of the scanner head, the finer and faster beam with regular profile manipulation is achieved, which is reflected by two galvo-mounted mirrors. The regularly spaced profile is taken perpendicular to the cutting direction. As shown in Figure 4b, the pattern drawn is a rectangle region with the width the same as the kerf width, and the length equal to a few millimeters, which is decided by the tube’s radius to obtain an available reaction. In order to maintain a deep cutting kerf, the multi-line scanner is used in our work, which avoids the screen of the laser beam. The pulse overlapping value is set to about 85% according to previous experiments [25]. Then, the laser beam is focused on the bottom of the groove in the *y* direction by an F-theta lens (shown in Figure 4a). The F-theta lens achieves a constant beam size across the entire scanning surface region. For the cutting process, the scanner head is used to move the laser beam to the groove’s bottom position, along with the rotary of the tube shell in low speed, to obtain the final dissection process.

### 2.2. Samples

The work-piece used in our research is a non-magnetic 304 austenitic stainless steel tube to imitate the neutron tube shell described in reference [8], which has an outer diameter of 25 mm, wall thickness of 2 mm, and length of about 160 mm. Table 2 presents the chemical composition of the 304 stainless steel (data were provided by the material supplier: Shanghai Baoshao Special Steel Co., Ltd., Shanghai, China), while the physical properties are presented in Table 3. During the dissection of the neutron tube shell, the inner domination temperature is required to be less than 50 °C and the temperature range of the shell body (the region 1 mm apart from the machining point) is required to be no more than 100 °C, while the residual high-power laser and its resultant molten slag are not allowed to enter the tube shell.

### 2.3. Numerical Modeling

**Figure 5:**
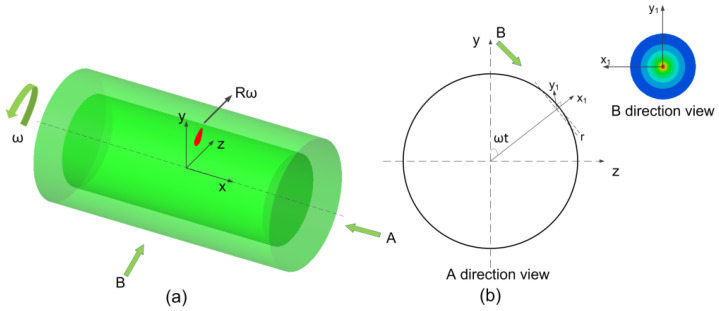
Schematic of the high-power fiber laser process FEM model: (**a**) axonometric view of FEM model; (**b**) direction views of FEM model.

To validate the temperature control of the shell surface during the CW laser dissection process, a three-dimensional finite element model (FEM) was established. The schematic of the dissection process using the CW laser is shown in Figure 5. The shell tube is set to be stationary due to relative motion, and the laser spot locates at the middle of the tube side surface and moves circularly along the tangential direction at a speed of 60 rad/s. The thermal effect of the light spot on the tube surface is related to the circular motion speed of the laser spot. The faster the circular motion, the shorter the time of laser action on the surface element and the slower the rate at which the surface temperature rises. The laser spot diameter is 100 μm and cuts at the outermost side of the tube, and the projection of the laser spot on the tube surface is an elliptical shape with a head and a tail as shown in Figure 5. The incident point of the laser on the tube changes with the tube rotation, as well as the local coordinate $\left(x_{1},y_{1}\right)$ of the laser spot, which is shown in the B-direction view in Figure 5b. The local coordinate origin of the laser spot on the tube surface can be expressed as $\left(\left(R-r\right)\sin\omega t\begin{array}{cc} , & \left(R-r\right)\cos\omega t \end{array}\right)$. The projection of the laser spot on the tube surface is realized by UDF (User Defined Function), and moves around the tube with the calculation time.

When the laser is directed on the surface of the shell tube, the target element surface is heated and the energy transfer includes heat conduction, heat convection, thermal radiation and so on. Besides, with the increase in temperature, melting occurs and is even followed by vaporization if the temperature reaches the boiling point. The laser flux density reaches 10^7^ W/cm^2^ in the dissection process; therefore, melting and vaporization are included in the calculation.

The governing equations for the conservation of energy can be expressed in the following form,

(1) $$\rho C_{pe}\frac{\partial T}{\partial t}-\nabla \cdot (k\nabla T)=Q-Q_{loss}$$

where ρ is the density of 304 stainless steel, $C_{pe}$ is the equivalent specific heat capacity, k is the heat conductivity, Q is the input laser energy flux density, and $Q_{loss}$ is the energy loss due to radiation and convection, which is written in the following equation,

(2) $$Q_{loss}=\sigma_{0}\varepsilon_{0}(T^{4}-T_{0}^{4})+h(T-T_{0})$$

where $\sigma_{0}$ is the Stefan–Boltzmann constant, $\varepsilon_{0}$ is the emissivity coefficient, T is the temperature of the shell tube surface, $T_{0}$ is the temperature of the surrounding environment, and h is the convective heat transfer coefficient. The flux density of laser energy follows a Gaussian distribution and can be expressed in the following form,

(3) $$Q=\frac{3A_{b}P}{\pi r^{2}}e^{-3\left[\frac{\left(x^{2}+y^{2}\right)}{r^{2}}\right]}$$

where $A_{b}$ is the absorption coefficient of the laser by 304 stainless steel, P is the injected laser power, r is the radius of the laser spot, and finally x and y are the coordinates in the Gaussian distribution. The energy deposition profile is a moving elliptical profile after projection on the tube surface through UDF.

In the FEM calculation, melting and vaporization are also included due to a high laser energy flux density of 10^7^ W/cm^2^ in the dissection process, and an equivalent specific heat capacity $C_{pe}$ is introduced, which is written in the following form,

(4) $$C_{pe}=C_{ps}+C_{m}D_{m}+C_{v}D_{v}$$

where $C_{ps}$ is the specific heat capacity of 304 stainless steel, $C_{m}$ is the latent heat of fusion, $C_{v}$ is the latent heat of evaporation, and finally $D_{m}$ and $D_{v}$ are Gaussian functions of fusion and evaporation, respectively, which can be written in the following form,

(5) $$D_{m}=\frac{\exp\left[-{\left(T-T_{m}\right)}^{2}/\Delta T_{m}{}^{2}\right]}{\Delta T_{m}\sqrt{\pi }},\;D_{v}=\frac{\exp\left[-{\left(T-T_{v}\right)}^{2}/\Delta T_{v}{}^{2}\right]}{\Delta T_{v}\sqrt{\pi }}$$

**Figure 6:**
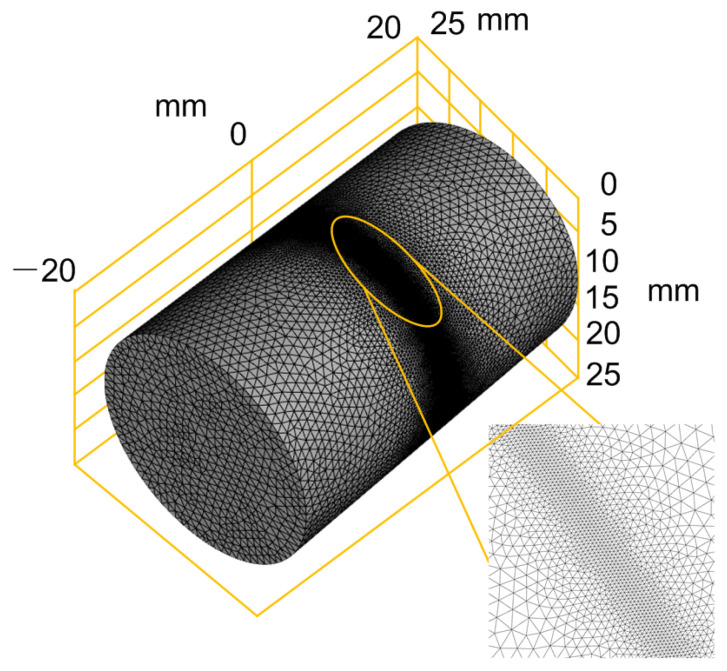
Component structure with mesh.

The finite element mesh model for the numerical calculation is shown in Figure 6, in which a cylindrical model with a length of 40 mm and an external diameter of 25 mm is established. The center area of the cylinder is the cutting area and grid refinement is adopted.

## 3. Results and Discussion

### 3.1. Numerical Modeling Results and Discussion

**Figure 7:**
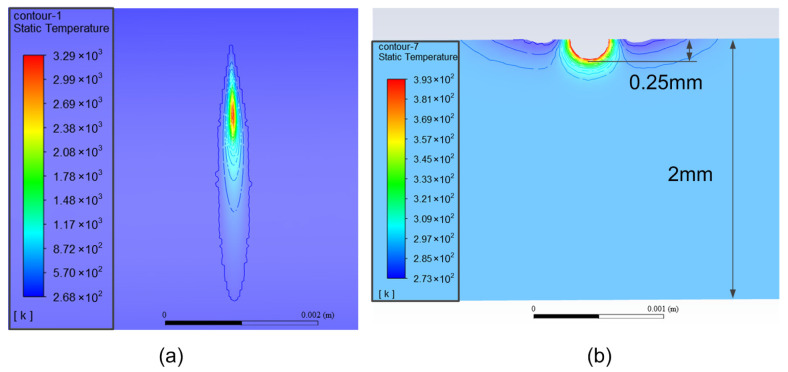
The contours of the temperature distribution: (**a**) temperature distribution near laser spot; (**b**) temperature profile in tube wall.

Figure 7 shows the contour of the temperature distribution on the tube surface and the profile of the tube wall as the calculation approaches a stable state. It can be seen from Figure 7a that the maximum temperature on the tube surface is about 3290 K. From Figure 7b, it can be seen that the temperature at the surface element irradiated by the laser is much higher and the temperature decreases rapidly from the outer wall to the inner wall. The depth of the area with a temperature higher than 100 °C is only 0.25 mm, which accounts for 1/8 of the total wall thickness, which shows that the cutting method proposed in this paper has little influence on the temperature inside the tube and meets the demand for controlling the temperature in the inner area of the tube during the dissection process. Due to the addition of jet airflow to remove residuals and for cooling the surface during dissection, a localized low temperature zone is formed around the high temperature zone, as shown in the profile view of the tube wall in Figure 7b, where the low temperature zone is located on both sides of the high temperature zone.

**Figure 8:**
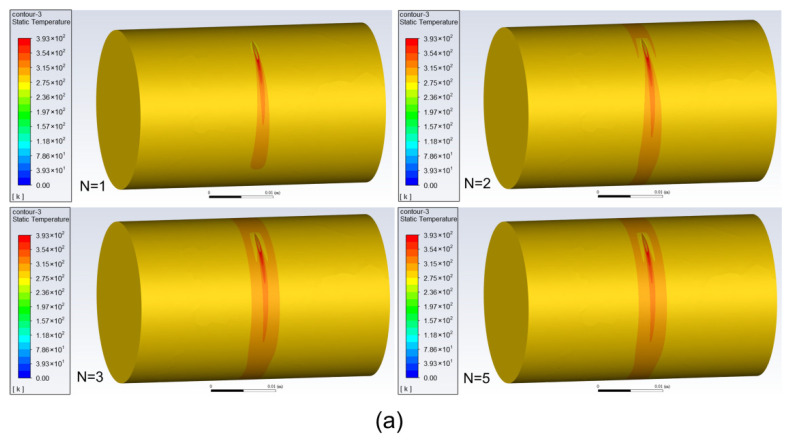

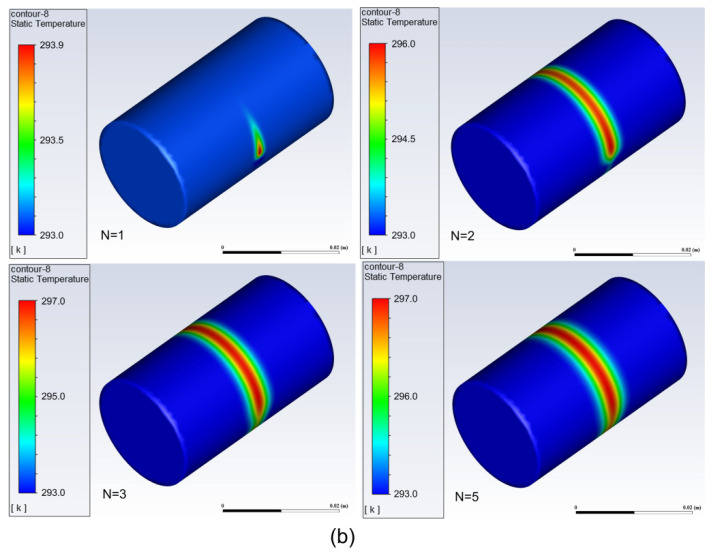
The contour of temperature distribution with different turns: (**a**) distribution on outer wall; (**b**) distribution on inner wall.

Figure 8 shows the contour of temperature distribution on the outer and inner walls of the tube with different turns of the laser moving around the tube. It can be seen that with the increase in turns, the high temperature areas on the outer and inner walls of the tube gradually expand, and after several turns, the temperature distribution approaches a stable state with a banding area of high temperature on the wall.

**Figure 9:**
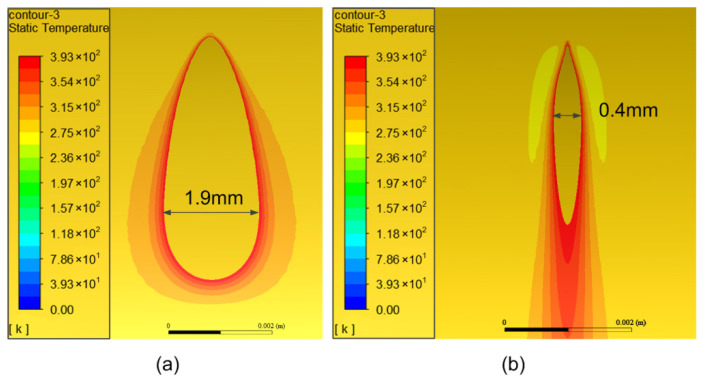
The area distribution of temperature beyond 100 °C: (**a**) rotating speed of 20 rad/s; (**b**) rotating speed of 60 rad/s.

Figure 9 shows the area distribution of temperature beyond 100 °C at different rotating speeds of the tube. It can be seen that when the tube rotates slowly, the laser irradiates the surface element for a longer time, and thus the temperature rises higher and the area of temperature exceeding 100 °C becomes wider. The maximum width of the temperature exceeding area is about 1.9 mm and 0.4 mm when the rotating speed is 20 rad/s and 60 rad/s, respectively. This means that an appropriate rotating speed is necessary to control the whole temperature distribution during the dissection process and the rotating speed adopted in this study was 60 rad/s.

### 3.2. Experimental Results and Discussion

**Figure 10:**
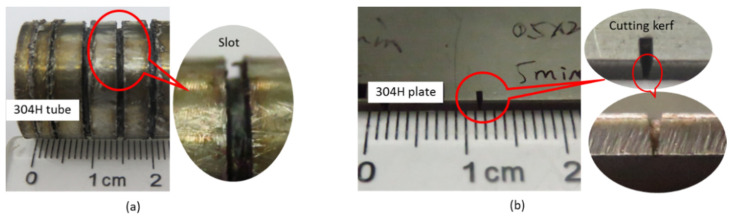
The results of cutting 304 stainless steel work-pieces: (**a**) the result of kilowatt fiber laser cutting of tube; (**b**) the result of 500-femtosecond pulse laser cutting of plate.

In this study, the dual laser beam asynchronous dissection process was divided into two machining experiments in order to certify the practicability of this new method. Firstly, a kilowatt continuous-wave fiber laser coupled to a laser cutting head was employed to manufacture a groove on the surface of a 304 stainless steel tube. The experiment parameters of this process were set to be the same as the first-step process of the new dissection method. Then, for the second experiment, a femtosecond pulse laser was used to produce a cutting kerf on a 304 stainless steel plate part. Both of them have the same material, i.e., 304 stainless steel. Figure 10 shows the results of cutting the tube. As shown in Figure 10a, a groove was successfully produced on the surface of the tube with a 25 mm diameter and a 2.5 mm wall thickness. The size of the slot was 1 mm width and 2 mm deep. It took about 1 min to produce the slot. Additionally, some black impressions could be seen on the surface of the tube near the slot, due to the influence of melt in the process. As shown in the previous Figure 2a, due to the high-pressure assist cutting gas, the molten slag is blown to the tangential direction of the tube. Figure 10b shows the experimental results of cutting the 304 stainless steel stainless plate with a thickness of 2 mm utilizing a femtosecond pulse laser. The whole process time of the plate was 5 min, the length of the cutting kerf was 1 mm and the width was 0.4 mm. The calculated cone half-angle of the cutting kerf was about 5.7°. The cutting speed was about 0.2 mm/min and the material removal rate was 0.2 mm^3^/min.

**Figure 11:**
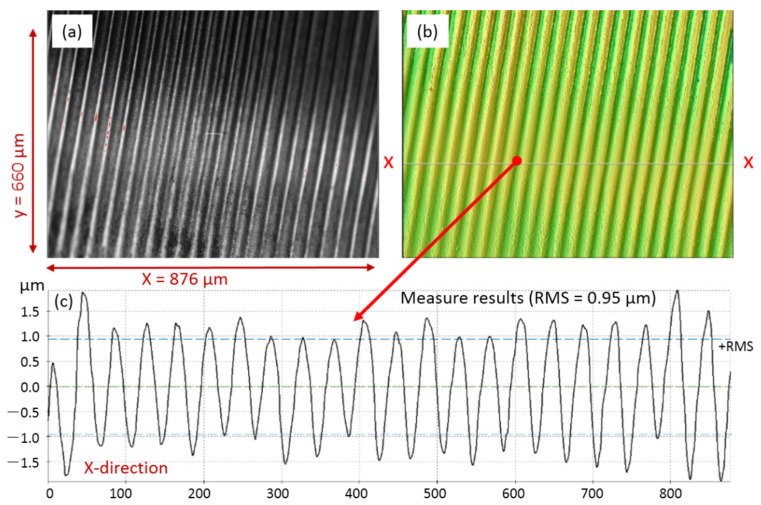
The side surface properties for the femtosecond pulse laser cutting kerf: (**a**) the side surface imaging formation of the cutting kerf wall; (**b**) the measured formation of the flank wall; and (**c**) the numerical value result for the x-x section.

For the result of femtosecond pulse laser cutting, the side surface wall properties inside the cutting kerf were imaged and measured by a confocal microscope (SensoSCAN neox, produced by Sensofar Metrology, Terrassa, Spain), as shown in Figure 11. As shown in the side surface imaging formation (Figure 10a), it was in the region of 876 μm length and 660 μm width, and it was a periodicity smooth plane without any molten slag on the flank wall. The measured results for the x-x section indicate that the surface roughness was about 0.95 μm. In addition, within the whole femtosecond pulse laser process, operating at room temperature, i.e., 25 °C, the monitored temperature was stabilized in the range of 38 °C to 41 °C. The results indicate that the thermal effect was avoided in the process.

Furthermore, with regard to the cutting of the neutron tube shell, with a size of 25 mm diameter and 2 mm wall thickness, which equals a 78.5 mm length plate, it needs 392.5 min (about 6.5 h). In addition, using the hybrid dissection method, after the first process steep of the high-power CW laser which needs 1 min, the remaining wall thickness to be cut is only 300 μm, and according to the cone half-angle of 5.7°, the cutting kerf width was less than 60 μm. This means that the material removal volume was smaller than 1.413 mm^3^ (0.3 mm × 0.06 mm × 78.5 mm). Consequently, with the same material removal rate, the required cutting time is just about 7 min. Adding the first steep time of 1 min, the total dissection time is about 8 min. That is to say, by the use of the hybrid dissection method, the cutting efficiency is improved about 49 times compared to only femtosecond laser cutting.

For the actual dissection processing of the neutron tube shell, all the cutting system is located inside a closed vessel which stays at a negative pressure. However, with the use of laser processing, it just needs an optic window to put the high-power laser beam transmission on the vessel, and an air pipe to facilitate the high-pressure cutting jet-flow inside the vessel. The exhaust gas generated in the process is gathered by a purification system. Additionally, there is a way to further cool the surface of the tube during the high CW laser cutting process by employing a second air jet-flow spraying up to the other side area which will be explored in our future work.

## 4. Conclusions

The sealed neutron tube shell dissection process utilizing the traditional lathe turning method requires frequent replacement of the diamond knife, which makes it a low-efficiency and high-expense approach. In this work, a novel dual laser beam asynchronous cutting method was presented to dissect sealed neutron tube shells with a material of non-magnetic stainless steel. Firstly, a high-power fiber laser is employed to produce a tapered groove on the surface of the tube shell, and then a femtosecond pulse laser is used to micromachine a cutting kerf at the groove’s bottom position.

A three-dimensional finite element model (FEM) was established to validate temperature control of the shell surface during the CW laser dissection process. It shows that the cutting method has little influence on the temperature inside the tube and meets the demand for controlling the temperature in the inner area of the tube during the dissection process. Due to the addition of jet airflow to remove residuals and cool the surface, a localized low temperature zone is formed around the high temperature zone. With the increase in turns, the high temperature areas on the outer and inner walls of the tube gradually expand, and after several turns, the temperature distribution approaches a stable state. When the tube rotates slowly, the laser irradiates the surface element for a longer time, and thus the temperature rise is higher and the area of temperature exceeding 100 °C becomes wider. The appropriate rotating speed is 60 rad/s to control the whole temperature distribution, with the depth of the area at a temperature higher than 100 °C equal to only 0.25 mm.

A kilowatt CW fiber laser was employed to make a groove on the surface of a 304 stainless steel tube with a 25 mm diameter and a 2.5 mm wall thickness. It took about 1 min to produce a slot of 1 mm width and 2 mm depth. Additionally, a 500-femtosecond pulse laser was used to produce a cutting kerf on a 304 stainless steel plate part with a thickness of 2 mm. It took 5 min to produce the kerf of 1 mm length and 0.4 mm width. The inner wall in the cutting kerf was smooth and without any molten slag. The monitored temperature was stabilized in the range of 38 °C to 41 °C, which indicates that the thermal effect is avoided in the process. By using the hybrid dissection method to cut a neutron tube shell of 25 mm diameter and 2.5 mm wall thickness, the cutting efficiency was improved about 49 times compared to the single femtosecond laser cutting.

According to the simulation and experiment results, this method provides a high-efficiency and non-thermal cutting technique for reclaiming neutron tube shells with advantages of non-contact, minimal thermal diffusion, and no effect of molten slag. Compared with the traditional fabrication methods, this technique provides an outstanding solution for the efficient and cold cutting of neutron tube shells, especially when the wall thickness is more than 1 mm. In summary, this work not only demonstrates an effective method for the cold cutting of steel tube shells with multi-millimeter level wall thicknesses, but also extends the application field of laser processing techniques.

## Figures and Tables

**Table 1:** Main parameters of the lasers.

Laser Parameters	CW Laser	Femtosecond Laser
Operation mode	Continuous wave	500 fs (Pulse)
Central wavelength	1080 nm	1030 nm
Average power	1000 W	7 W
Repletion rate	N/A	300 kHz
Focal length	150 mm	100 mm
Beam diameter	100 μm	30 μm
Beam mode	TEM_00_ Gaussion Mode	

**Table 2:** Chemical composition of 304 stainless steel for tube shell with a 25 mm diameter [26].

Type	Mass Fraction	C	Si	P	S	Mn	Ni	Cr
Tube shell	Max (%)	0.10	0.75	0.04	0.03	2	11	20
	Min (%)	0.04	-	-	-	-	8	18

**Table 3:** Physical properties of 304 stainless steel.

Material Properties	Symbol	Value
Density	ρ	7200 (kg·m^−3^)
Thermal conductivity (solid/liquid)	k	40/22 (W·(m·K)^−1^)
Specific heat capacity (solid/liquid)	$C_{ps}$	720/800 (J·(kg·K)^−1^)
Coefficient of thermal expansion	δ	4.95 × 10^−5^ (K^−1^)
Fusion latent heat	$C_{m}$	2.47 × 10^5^ (J·kg^−1^)
Evaporation latent heat	$C_{v}$	6.34 × 10^6^ (J·kg^−1^)
Solid temperature	$T_{s}$	1679 (K)
Liquid temperature	$T_{l}$	1727 (K)
Fusion point	$T_{m}$	1700 (K)
Boiling point	$T_{v}$	3200 (K)
Emissivity coefficient	$\varepsilon_{0}$	0.16
Convective heat transfer coefficient	h	40 (W·m^−2^·K^−1^)
Stefan–Boltzmann constant	$\sigma_{0}$	5.67 × 10^−8^ (W·m^−2^·K^4^)
